# Exercise in Myositis

**DOI:** 10.1007/s40674-018-0113-3

**Published:** 2018-11-23

**Authors:** Helene Alexanderson

**Affiliations:** Department of NVS, Huddinge, Karolinska Institutet and Department of Medicine, Karolinska Institutet, Solna and Function Area Occupational Therapy and Physical Therapy, Karolinska University Hospital, SE-171 76 Stockholm, Sweden

**Keywords:** Dermatomyositis, Exercise, Inclusion body myositis, Polymyositis

## Abstract

**Purpose of the review:**

A growing body of evidence supports exercise as a very important part of the treatment for adult patients with idiopathic inflammatory myopathies (IIM). This review mainly focuses on exercise studies published during the last 2 years in adult myositis.

**Recent findings:**

During the last couple of years, new publications present further evidence for intensive endurance exercise as an anti-inflammatory treatment inducing muscle growth and improving mitochondrial function compared a non-exercising control group. Further, blood-flow restricted resistance training was effective to maintain muscle strength compared to a non-exercising control group which lost a mean of 9.2% in quadriceps strength over 3 months in inclusion body myositis. Another study evaluates the effects of intra-muscular injections of an isoform of follistatin (FS344) by AAV1 in combination with exercise in a small group of patients with inclusion body myositis. An improvement in physical capacity was associated to higher exercise levels. Less is known about exercise effects in patients with recent onset, active polymyositis, and dermatomyositis.

**Summary:**

All studies report safety of exercise in all types and stages of myositis and exercise could now be considered as medicine. It is recommended to initiate exercise on a low intensity under supervision of a physical therapist with regular follow-up and progression of intensity over time.

## Introduction

### The idiopathic inflammatory myopathies and effects of exercise

The inflammatory myopathies are a heterogenous group of disorders divided into sub-groups adult polymyositis (PM), dermatomyositis (DM), and inclusion body myositis (IBM), as well as the juvenile dermatomyositis (JDM). Muscle impairments are the cardinal symptom in IIM, but involvement of extra-muscular organ systems is common, such as interstitial lung disease (ILD) and skin rash in DM. Patients with PM and DM present with reduced muscle strength, but more so with reduced muscle endurance [1]. Despite an often favorable effect of medical treatment, most patients develop sustained disability and reduced quality of life [2, 3]. Patients have reduced aerobic capacity compared to healthy, matched controls [4, 5]. Fatigue, pain, and cognitive impact are other important disease consequences that have not been much addressed in the literature [6].

Over the last two decades, many studies have been conducted to investigate safety and effects of exercise in adults with myositis, with the majority focusing on polymyositis and dermatomyositis [5, 7–33, 34••, 35•, 36••]. The emerging evidence supports exercise as a safe and effective treatment to optimize health and reduce disability in adult patients with myositis. Excitingly, intensive exercise can even reduce disease activity and inflammatory markers as well as to improve muscle tissue health in adult polymyositis and dermatomyositis [4, 16, 32, 34••, 37]. Exercise has become an important part of the treatment in both adult and juvenile IIM. This review will mainly focus on studies published during the last 2 years in adult patients with IIM.

### Effects of exercise in adult PM/DM

#### Disease activity, inflammation, and muscle health

Two studies have reported on effects of exercise on disease activity and inflammation in adults with polymyositis and dermatomyositis. Two of eight patients with established polymyositis and dermatomyositis were responders with reduced disease activity [16] according to the established criteria (> 20% improvement in > 3/6 variables, with worsening > 20% in no more than two variables which cannot include the Manual Muscle Test, MMT) [38]. Micro array analysis of muscle biopsies pre- and post-training revealed downregulation of genes related to inflammation and fibrosis compared to baseline [19]. A randomized controlled trial evaluating an intensive aerobic and endurance-based exercise program supported the hypothesis that intensive exercise does not only improve objective and patient-reported function and quality of life, but also can reduce disease activity in established PM and DM [5, 32]. The program consisted of 30 min of stationary biking on 70% of maximal oxygen uptake VO_2max_ together with endurance-based resistance training performed 3 days a week for 12 weeks. This type of exercise induced several positive changes in clinical disease activity both on a group level and, according to the responder criteria, and improved muscle metabolism that have been reviewed elsewhere [39, 40]. New publications report on changes in inflammatory activity and in muscle health that could explain the improvements in physical capacity and quality of life by this intensive aerobic exercise program (Table 1).

**Table 1:** Effects of exercise on disease activity, inflammation, metabolism, and muscle characteristics in patients with adult myositis (exercise studies published during 2016–2018)

Study/design	Diagnosis/patients, *n*/disease activity HC, *n*	Exercise/duration/frequency	Load/intensity, % of max/VRM	Disease activity compared to HCs where applicable/responders, *n*	Physical capacity/self-reported outcomes	Inflammation and gene expression/metabolic changes
Alemo Munters et al. 2016/RCT [36••]	PM, DM EG *n* = 6 CON *n* = 7 established	Aerobic, endurance/12 weeks/3 days/week	70%/VO_2max_/1 × 30–40 VRM	6-Item core set EG: Resp. *n* = 5 CON: Resp. *n* = 1	Muscle endurance (biking test to exhaustion on 70%/max: EG: resp. *n* = 6 CON: resp. *n* = 0 VO_2max_: EG: resp. *n* = 4 CON: resp. *n* = 0	EG (altered gene expr. in 270 genes) Upregulation: capillary growth, mitochondrial biogenesis, protein synthesis, cytoskeletal remodeling, muscle hypertrophy. Downregulation: inflammation/immune response and endoplasmic reticulum stress. Higher number of capillaries per mm^2^ (*P* < 0.05). CON (altered expr. In 227 genes) Upregulation: IFN signaling, apoptosis
Jörgensen A et al. 2017/case report [35•]	IBM, *n* = 1	Blood-flow restricted quadriceps resistance training, cuff pressure 100 mmHG/12 weeks	25 VRM/4–5 45 s bouts	CK: unchanged	Quadriceps IMVC: + 60% Quadriceps CPM: unchanged knee AROM: + 68% 10-m max gate speed: + 19% TUG: Unchanged	–
Jörgensen A et al. 2018/RCT [36••]	IBM, *n* = 22 BFR, *n* = 11 CON, *n* = 11	Blood-flow restricted resistance quadriceps training, cuff pressure 110 mmHG/12 weeks	25 VRM/4–5 45 s bouts	MDAAT: Pat. Global BFR/CON: unchanged Phys. Global BFR: reduced (*p* = 0.047) CON: reduced (*p* = 0.004) CK BFR: unchanged CON: reduced (*p* = 0.04) HAQ Pat. Global BFR/CON: unchanged MMT Pat. Global BFR/CON: unchanged MDI Pat. Global BFR: reduced (*p* < 0.001) CON: unchanged Phys. Global BFR: reduced (*p* = 0.03) CON: reduced (*p* < 0.001)	SF-36 PF: no between or within group differences IBMFRS: BFR: + 3.7%/CON: − 2.1 between-group (*p* = 0.018) Quadriceps strength: BFR: + 5.8% (*p* = 0.30) CON: − 9.2% (*p* = 0.02)	

*PM* polymyositis, *DM* dermatomyositis, *IBM* inclusion body myositis, *HCs* healthy controls, *EG* exercise group, *CON* control group, *BFR* blood-flow restricted, *VO*_*2max*_ maximal oxygen uptake, *VRM* voluntary repetition maximum, *IMVC* isometric voluntary contraction, *CPM* concentric power maximum, *AROM* active range of motion, *MDAAT* myositis disease activity assessment tool, *Pat. Global* patient global assessment, *Phys. Global* physician global assessment, *CK* creatine phosphokinase, *HAQ* health assessment questionnaire, *MMT* manual muscle test, *SF*-*36 PF* SF-36 physical function, *IBMFRS* inclusion body myositis functional rating scale, *IFN* interferon, *TUG* timed-up and go

Pre- and post-exercise muscle biopsies were available for a sub-group of patients from this study. The intensive exercise program induced a downregulation of protein degradation and in genes related to inflammation, immune response, and ER-stress. In addition, upregulation of genes related to muscle growth and signs of capillary growth. No similar response was identified in the non-exercising control group [34••]. Analysis of a smaller sub-set of patients from the RCT by Alemo Munters et al. (2013) [32] revealed changes in microRNAs targeting transcripts and proteins that are important for immune and muscle response [41••]. These studies further support the hypothesis that exercise is a disease-modifying treatment and an important part of the treatment in patients with PM and DM.

#### Disability and quality of life

The intensive aerobic exercise as described by Alemo 2013 et al. [32] significantly improved aerobic capacity and muscle function compared to the non-exercising control group [4, 32]. As expected, the aerobic biking program and the muscle endurance training resulted in foremost improved aerobic capacity assessed as the (VO_2max_) and the biking time to exhaustion on 70% of maximal effort [4]. The exercise group also improved significantly in patient-reported physical function and vitality (SF-36) compared to the control group and there was additional within–exercise group improvement in SF-36 General Health and Mental health while the control group was unchanged. VO_2max_ and patient-reported physical function correlated strongly both at baseline and after 12 weeks of exercise indicating that aerobic capacity is an important variable controlling patient-reported health. Quadriceps muscle strength was also improved in the exercise group as assessed by the five voluntary repetition maximum (the weight the patient can lift a maximal of five times), compared to the control group and interestingly, this variable was the only to be consistently improved in the exercise group at the one-year follow-up, while all other variables returned to baseline values. Alemo et al. also investigated the effects of exercise on activity limitations (ability to perform daily activities). Self-reported ability to walk, climb stairs, and run (myositis activities profile sub-scale moving around) was significantly improved in the exercise group compared to the control group with additional exercise group improvement in sub-scales social activities and leisure activities. In addition, patient-preference activities were also improved by exercise in the exercise group. According to available evidence in patients with established polymyositis and dermatomyositis, exercise recommendations for patients with myositis do not differ significantly from established recommendations of exercise to improve muscle function and aerobic capacity (Table 2).

**Table 2:** Recommendations for health-enhancing physical activity and exercise for healthy individuals [48]

	Duration of exercise bouts, min	Intensity, percentage of VRM	Intensity, percentage of age predicted maximal heart rate	Frequency, times/week
Increase muscle strength	–	60–80	–	2–3
Increase muscle endurance	–	30–40	–	2–3
Increase aerobic capacity	30–60	–	60–85	3
Improve/maintain health	30	50–70	–	4–7

*VRM* voluntary repetition maximum

Only a few studies have included patients with recent onset, active polymyositis, and dermatomyositis and no study with this focus has been published during the last 2 years. A home exercise program on low or moderate intensity (adapted to individual disease activity and disability) has been extensively investigated in patients with recent onset disease [12]. As the program can be individually adapted, it is well tolerated by all patients. The program was evaluated in combination with moderate-intensity walks 5 days a week in a randomized controlled trial supporting the safety but was not able to show additional short-term positive effects on muscle function and aerobic capacity in combination with standard medical treatment compared to a control group receiving medical treatment alone [33]. However, the exercise group was at 1 and 2 years follow-up more physically active and had improved physical capacity compared to the control group. This implies that it is important to start exercise early to have a physically active lifestyle and improved physical capacity later in the disease course. The hypothesis is that a more intensive exercise program is needed to achieve improved physical capacity and quality of life in addition to the medical treatment. In background of new knowledge of the anti-inflammatory effects of exercise proven in patients with an established myositis, it is of outmost importance to focus research on safety and effects of exercise early in the disease course. A randomized controlled trial investigating clinical and molecular effects of high-intensity interval training is ongoing at the Karolinska University Hospital (clinicaltrial.gov ID NCT03324152).

### Exercise effects in sIBM

Two case reports exploring feasibility of blood-restricted resistance training (BRF) in sporadic inclusion body myositis indicated positive effects on muscle strength [22, 35•]. A randomized controlled trial from group in Denmark was not able to show improvement in self-reported or objectively assessed function in the exercise group compared to the control group [36••]. However, there was a striking significant time effect difference regarding knee extensor muscle strength in favor for the exercise group which was unchanged (+ 5.8%) while the control group worsened (− 9.2%). This time effect difference was also true for the self-reported physical function assessed by the IBM functional rating scale indicating that exercise slows down disease progression and maintains function and physical independence. Individuals with IBM were randomized either to the BRF group exercising twice a week for 12 weeks or to a non-exercising control group. The BRF group performed supervised unilateral knee extensions in a leg press in three sets of 25 VRM, allowing 25 repetitions until exhaustion with a fourth set introduced at week 9. Four weeks into the exercise program, knee flexion exercises were introduced according to the same protocol. The program also contained calf raise and dorsiflexion of the ankles. Blood flow was restricted by inflicting a 100 mm wide inflatable pneumatic cuff to 110 mmHG, connected to a computerized tourniquet system. The authors discussed that the exercise period of 12 weeks might have been too short to be able to achieve improvements and that the study was slightly under-powered. In addition, there were within-patient differences in exercise response. Patients with less disease duration had a better response indicating the importance of introducing exercise as a treatment early in the disease course with a possibility to postpone damage and disability. Authors discuss further that the reduced disease activity seen in both groups was likely related to interrater variability and not a result of the intervention. The 16-week home exercise program that was evaluated in an open-design study including seven patients with IBM did achieve significant and clinically relevant improvements in knee extensor strength and functional tests such as 30-m walk and sit-to-stand test [30]. Clinical experience from using this program at the Karolinska University Hospital supports these positive results to some extent. Similarly, to the RCT by Jörgensen et al., clinical experience indicates that some patients with IBM respond very well to treatment, improving both in strength and functional capacity, while the large majority experience small improvements in one functional test. However, as reported by Jörgensen et al. [36••] and based on clinical experience, few patients might not tolerate exercise well with increased muscle weakness or increased pain as a result of exercise. Today, we are not able to predict which patients with IBM will tolerate and respond to exercise or not, and a relevant question is if immunosuppressive treatment increases the chances to respond to treatment? Furthermore, there is an urgent need for larger RCTs evaluating different exercise programs to understand what type of exercise is the most efficacious and what factors might predict response to exercise. Recently, a large RCT investigated the effects of the myostatin inhibitor Bimgrabumab in IBM [42]. Primary outcome was the 6-min walking test, which was not altered compared to the placebo-controlled group and the study was stopped after 1-year follow-up. Levels of exercise or physical activity among participants in both groups were not registered. Mendell et al. [43•] treated five patients with IBM with intra-muscular injections of an isoform of follistatin (FS344) by AAV1 in combination with exercise [43•]. All patients were encouraged to exercise as much as possible but to do a minimum of 15-min stationary biking and knee extensor resistance training 3 days a week for the study period of 1 year. At 1-year follow-up, the treatment group was significantly and clinically relevant improved in the primary outcome 6MWT compared to a non-treated control group matched for age, disease duration, and baseline 6MWT. Further, a larger improvement in 6MWT was associated to regular exercise habits. Based on this, it would be interesting to test Bimgrabumab in combination with a specific exercise program. As of today, we do not know what exercise regimen is most effective in IBM; however, evidence supports safety of individually adapted exercise and that regular exercise can maintain function while inactivity leads to reduced muscle function [36••].

### Specific hand exercise programs in all adult patients with myositis

It is well-known that a progressing, severe muscle weakness of finger and wrist flexors is a cardinal symptom in inclusion body myositis. An ongoing hand exercise study at the Karolinska University Hospital in Stockholm will hopefully soon improve our understanding on how individualized specific hand exercise can improve grip strength and hand function in patients with inclusion body myositis. Grip strength is also reduced in patients with adult polymyositis and dermatomyositis compared to reference values (2) and a specific hand exercise program could improve both grip strength and dexterity in these patients (regard hand exercise). Individualized hand exercise is therefore also an important part of the treatment in myositis and should be initiated ideally by an occupational therapist with knowledge of myositis.

### Discussion

During the last 2 years, a few new publications have emerged improving our understanding of the effects of exercise on muscle health and inflammation. Exercise can improve mitochondrial function, angiogenesis as well as improve muscle growth and reduce inflammation in established polymyositis and dermatomyositis. Further, a randomized control trial was recently published that strongly supports the importance to exercise with the purpose to maintain or in some cases also improve muscle function in patients with inclusion body myositis.

Exercise reduces systemic inflammation in healthy individuals [44] and studies in other inflammatory conditions such as rheumatoid arthritis and systemic lupus erythematosus also indicate less inflammation by exercise [37]. Thus, it is not surprising that intensive exercise has shown the same positive effects in patients with polymyositis and dermatomyositis. The notion that exercise could be harmful in myositis was based on studies in healthy athletes performing strenuous types of exercise such as a Marathon race [39]. An acute response with increased serum creatine phosphokinase levels (CK) is normal and levels go back to baseline levels within 24 h. There will also be an intra-muscular inflammatory response, with increased IL6-levels, which is part of the regeneration leading to improved muscle function [37]. All exercise studies in myositis report unchanged CK levels over weeks as well as unchanged or somewhat lower inflammation after exercise assessed by MRI or analysis of muscle biopsies [11, 12, 16, 32] in both established and early, active disease. Thus, individually adapted exercise should be introduced within weeks from starting medical treatment with frequent follow-up, at least every third month, during the first year. Exercise also needs to be progressed regularly according to changes in disease activity and physical capacity.

A recent study reports mitochondrial abnormalities in patients with recent onset untreated dermatomyositis with increased reactive oxygen species (ROS) production and interferon type I signature in muscle tissue correlating to reduced exercise capacity [45]. These data support introduction of exercise early in the disease course to counteract muscle pathology in muscle and hopefully restoring muscle function and muscle health sooner and to a larger extent than today. In addition, exercise could reduce corticosteroid-induced muscle atrophy and osteoporosis. However, more studies are needed.

The few studies of exercise effects in inclusion body myositis as well as clinical experience indicate that these patients might need to exercise more frequently to be able to achieve clinically meaningful improvements in the most affected muscle groups (Johnson et al. 2007), than patients with polymyositis or dermatomyositis. The study by Jörgensen et al. 2017 [36••] is very important as the first study to describe the natural course of deterioration of muscle weakness compared to twice a week exercise in inclusion body myositis, strongly supporting the role of exercise as a treatment to maintain and possibly also improve muscle strength in these patients. Only one case report has included analysis of muscle biopsies before and after blood-flow restricted resistance training in one patient with inclusion body myositis revealing slightly increased muscle fiber area and no signs of increased inflammation or damage [31]. Further research is needed to understand muscle pathology in inclusion body myositis and how exercise could improve muscle health in these patients.

The concept to use improvement criteria to study individual exercise response is interesting. Myositis is a heterogeneous disease where many factors can influence exercise response. Tolerance and response to medical treatment and the disease duration until diagnosis and subsequent degree of damage are two factors that may influence exercise response. Further, different antibody profiles and different disease mechanisms probably also play a role. The first improvement criteria for disease activity were proven useful to identify individuals responding to exercise with reduced disease activity [16, 32]. In addition, the ACR 20, 50, and 70% responder criteria [46] could identify patients responding in muscle function, aerobic capacity, and activity limitations by exercise [16, 32]. In addition, new revised response criteria for disease activity were recently published [47]. Using these criteria in addition to group analysis will enhance individualized treatment by understanding what patients respond to different types of exercise. Clinical experience suggests a variation in exercise response in patients with inclusion body myositis. Again, diagnosis duration and degree of damage are two important factors, but there are probably different subtypes of inclusion body myositis with varying muscle involvement and progression time and knowledge of the roles of antibodies is very limited. Our own clinical experience indicates that most patients tolerate exercise well with limited improvements or mostly maintaining function. A few patients seem to respond very well with larger improvements in function and tolerating a very high exercise frequency and intensity, while yet a smaller group of patients might not tolerate even mild exercise. There is an urgent need for more, larger studies looking into exercise in inclusion body myositis.

In summary, exercise is a very important part of the treatment in adult patients with myositis to optimize function and quality of life as well as disease activity. Patients with recent onset disease require regular follow-up and adaption of exercise to changes in physical capacity, fatigue, and pain and exercise should ideally be initiated and followed up by a physical therapist with knowledge of myositis. Specific hand exercise is also helpful to optimize function in patients with myositis. Myositis is a multi-organ system, heterogeneous disease and thus requires an expert team of medical and rehabilitation health professionals to optimize prognosis and quality of life.
